# Sac enlargement due to perigraft seroma and back-bleeding from the remnant wall 11 years after open surgical repair of an infected abdominal aortic aneurysm

**DOI:** 10.1016/j.jvscit.2022.01.006

**Published:** 2022-02-08

**Authors:** Kazumasa Hanada, Katsuyuki Hoshina, Masamitsu Suhara, Ryosuke Taniguchi, Mitsuru Matsukura, Toshio Takayama

**Affiliations:** Division of Vascular Surgery, Department of Surgery, Graduate School of Medicine, The University of Tokyo, Tokyo, Japan

**Keywords:** Perigraft seroma, Perigraft hygroma, Endoleak, Abdominal aortic aneurysm, Complication

## Abstract

We describe a case of sac enlargement that occurred 11 years after emergent open surgical repair of an infected abdominal aortic aneurysm. The diameter of the sac covering the Dacron graft had gradually expanded to 80 mm, and the flow of contrast medium into the sac was suspected. Elective surgery revealed a perigraft seroma and back-bleeding from the remnant wall. After attaining hemostasis, fibrin glue and oxidized cellulose were applied, and sac plication was performed. Thereafter, the sac has not expanded. Open diagnostic treatment should be a good option for cases of postoperative sac enlargement with an unknown origin.

Sac dilation after open abdominal aortic aneurysm (AAA) repair has been reported and is mainly caused by perigraft seroma (PGS) or hygroma; additional causes include infection and bleeding.^1^, ^2^, ^3^, ^4^, ^5^, ^6^, ^7^, ^8^ A PGS is a rare complication defined as a persistent, sometimes expanding, sterile fluid collection within a nonsecretory, fibrous pseudomembrane surrounding a patent vascular graft.^1^ Herein, we describe a case of sac enlargement occurring 11 years after open AAA repair, which resulted from PGS and back-bleeding from the remnant wall. The patient consented to publication of the details and images pertaining to the case.

## Case report

**Fig 1:**
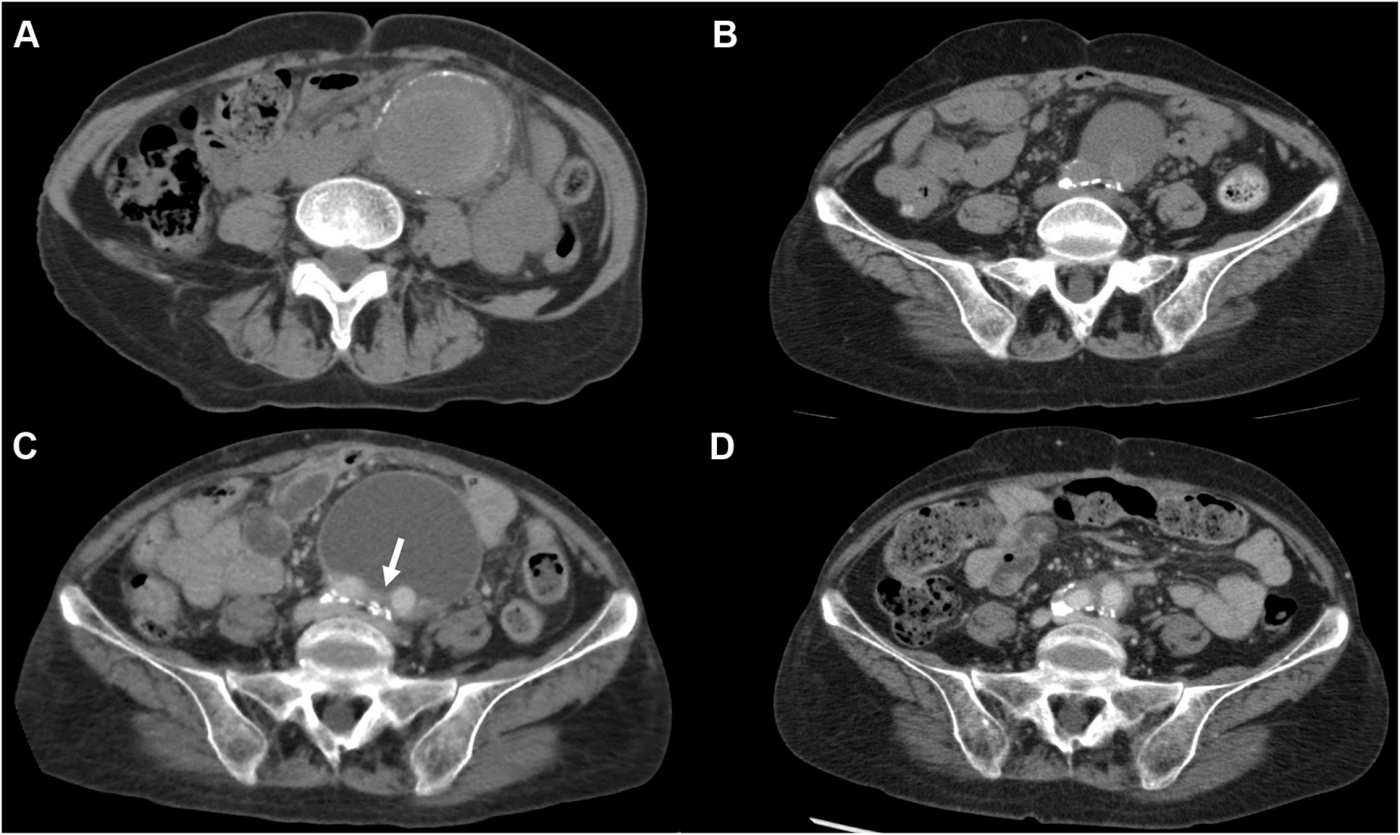
**(A)** Computed tomography (CT) scans showing an impending rupture of a 54-mm infected abdominal aortic aneurysm (AAA). **(B)** CT scans showing a 40-mm sac covering the graft 9 years after repair of the aneurysm. **(C)** Contrast-enhanced CT scans showing an 80-mm sac filled with low-density fluid around the graft. The white arrow indicates the contrast medium flowing into the sac. **(D)** Contrast-enhanced CT scans showing a small amount of fluid around the graft and no contrast medium flowing into the sac (11 years after repair of the aneurysm and 3 years after laparotomy).

A 77-year-old woman with slight abdominal discomfort was admitted to our department for investigation and treatment of an 80-mm sac. She had undergone open AAA repair 11 years ago for impending rupture of an infected AAA (Fig 1, *A*). A rifampicin-soaked Dacron bifurcated graft (GELSOFT PLUS, 16 × 8 mm; Vascutek, Inchinnan, Scotland) was placed in situ, and the aneurysmal wall excepting the posterior wall was removed. The graft was wrapped with the omentum and the posterior wall. No causative microorganisms were detected in the cultured specimens or pus. Although the patient’s chronic kidney disease worsened postoperatively, she was discharged uneventfully. One year later, hemodialysis was started. Five years after the index operation, follow-up computed tomography (CT) findings revealed the presence of a 40-mm sac, and a diagnosis of PGS was made (Fig 1, *B*). The radiodensity of the collected fluid did not exceed 30 Hounsfield units. For the next 4 years, the sac, including the graft, was stable. Nine years after the index operation, coronary artery bypass surgery was performed for angina, and anticoagulant therapy was started for atrial fibrillation. In the following 2 years, the sac grew by approximately 20 mm/year, ultimately reaching 80 mm in diameter.

On physical examination, the patient was 152 cm tall and weighed 45.4 kg (body mass index, 19.9 kg/m^2^). A mass in the lower abdomen was palpable but not pulsatile. Owing to arteriosclerosis, the pedal pulses were poor. The routine blood test results were unremarkable, and there were no clinical findings of inflammation or infection. The patient’s general condition was sufficient for open surgery. Contrast-enhanced CT scans showed that the sac was filled with low-density fluid, with the contrast medium flowing into the sac in the late phase (Fig 1, *C*). We suspected that the sac enlargement was caused by back-bleeding from the posterior wall, PGS, or infection. We opted to perform elective open surgery, considering the graft replacement, if necessary, because of the presence of abdominal discomfort, sac enlargement, bleeding, and risk of rupture. We did not select a percutaneous drainage, which could have led to the recurrence of PGS. Endovascular therapy is not considered appropriate in cases where the cause of the sac dilation is infection and where the main content of the sac is not blood; therefore, coagulation after embolization was not expected. Moreover, the patient had no proper access sites because of severe arteriosclerosis.

**Fig 2:**
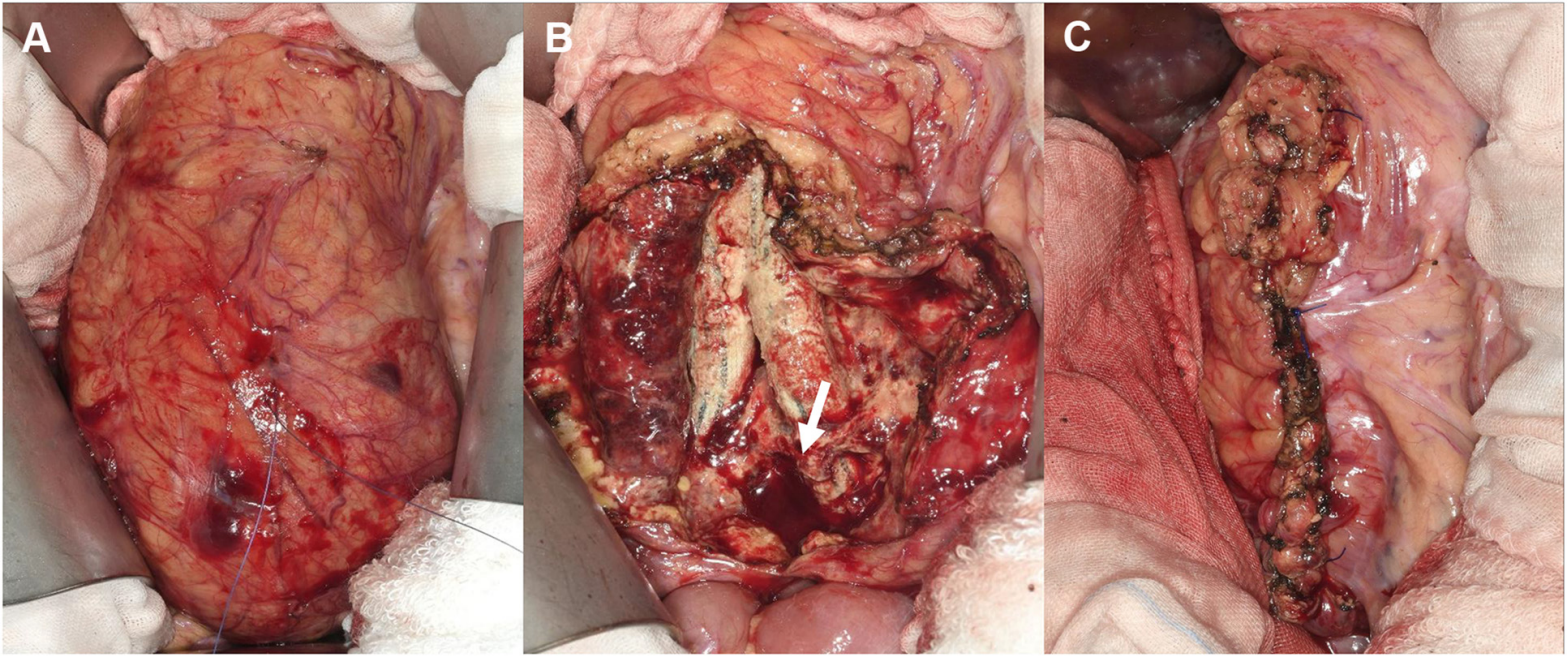
Intraoperative photographs showing **(A)** the 80-mm soft and nonpulsatile sac, **(B)** the intact Dacron graft encased in a gelatinous material and back-bleeding from the sac and posterior wall (arrow), and **(C)** the plication of the sac.

A midline laparotomy was performed, and the intra-abdominal adhesions were separated. The sac was soft but not pulsatile (Fig 2, *A*). Puncturing of the sac released 200 mL of a slightly turbid, straw-colored fluid followed by hemorrhagic fluid, resulting in the collapse of the sac. We incised the sac without clamping the aorta. A gelatinous material on the surface of the graft and minor bleeding from the omentum and posterior wall surrounding the graft were observed (Fig 2, *B*). The graft was not dilated or attached to the omentum or posterior wall. There was no sign of bleeding at the anastomoses or extravasation through the graft. Our diagnosis was PGS accompanied by minor bleeding from the remnant sac, and graft replacement was considered unnecessary. After achieving hemostasis via cauterization and ligation, fibrin glue and oxidized cellulose were applied around the graft (Video). Subsequently, resection and plication of the sac were performed (Fig 2 *C*). The blood loss was 130 mL, and the operating time was 131 minutes.

Cytological examination indicated bleeding and inflammation. The cultures of the fluid sample were negative. Contrast-enhanced CT findings on postoperative day 4 confirmed the shrinkage of the sac and the absence of contrast medium entry into the sac. The patient was discharged on postoperative day 11 without the abdominal discomfort. There was no sac dilation found at 3 years after the laparotomy (Fig 1, *D*).

## Discussion

The causes of postoperative sac enlargement include PGS, infection, anastomotic aneurysms, transinterstitial bleeding through the graft, and minor bleeding from the sac.^1^, ^2^, ^3^, ^4^, ^5^, ^6^, ^7^, ^8^ The last three resemble type I, IV, and II endoleaks, respectively, and a PGS resembles a type V endoleak or endotension in the endovascular aortic repair era. Sac enlargement can cause rupture and acute limb-threatening ischemia owing to graft thrombosis.^2^^,^^3^^,^^9^, ^10^, ^11^

Seromas have been reported in patients with polytetrafluoroethylene (PTFE) and Dacron grafts; those after open AAA repair are mainly attributed to the PTFE grafts (approximately 20% of the expanded PTFE grafts) instead of the Dacron grafts (0%-3.8%).^1^, ^2^, ^3^ The cause of PGS remains unclear, but involves inadequate graft healing or incorporation and/or transgraft passage of fluid from the aortic lumen to the sac.^2^, ^3^, ^4^^,^^9^ Factors associated with PGS development after open AAA repair include diabetes, smoking, anticoagulation, bifurcated graft reconstruction, large graft diameter, and the use of a left flank retroperitoneal approach for repair.^2^^,^^3^ Intervention should be considered in patients with symptoms or a PGS of greater than 8 cm to avoid life- or limb-threatening complications; small asymptomatic seromas do not require intervention.^3^ The most effective, although invasive, treatment is replacement of the entire graft with an alternative material; other treatments include relining the graft with covered stents, drainage, resection and plication using the omentum, and the application of fibrin glue.^2^^,^^3^^,^^9^, ^10^, ^11^, ^12^, ^13^, ^14^, ^15^, ^16^ The use of several procedures in combination has been reported.^12^^,^^13^ Simple aspiration can lead to the recurrence of PGS.^2^^,^^3^

In our case, PGS was diagnosed before admission because the radiodensity of the fluid within the sac was low and the sac had been stable.^2^^,^^3^ The Dacron graft and the omentum did not prevent PGS development. Sac dilation occurred after anticoagulant therapy initiation, and enhanced CT findings revealed bleeding within the sac. Anticoagulation might contribute to bleeding from the posterior aneurysmal wall and the omentum as well as enlargement of a PGS.^17^^,^^18^ Intraoperative findings, such as the straw-colored fluid in the sac and the gelatinous material on the surface of the graft, supported the diagnosis of PGS.^9^, ^10^, ^11^ Bleeding from the posterior aneurysmal wall and the omentum was minor and difficult to detect preoperatively. Aortography could have revealed the blood flow from the posterior wall, but not from the omentum. Owing to the possibility of infection, we did not use an endograft, perform percutaneous aspiration, or inject sclerosing agents, each of which might have shrunk the sac. Open surgery was considered appropriate. The application of fibrin glue and oxidized cellulose might promote graft incorporation and prevent PGS recurrence.

## Conclusions

It should be considered that sac enlargement after open AAA repair can involve not only PGS, but also minor bleeding from the sac. In this endovascular aortic repair era, follow-up examinations after open AAA repair should include contrast-enhanced CT or duplex ultrasound examination to rule out any endoleak-like events. Open surgery or endovascular therapy can be selected according to the symptoms, size of the sac, and mechanism of sac enlargement.

## Appendix

Video The duodenum was separated from the sac. The sac was soft but not pulsatile. Puncturing the sac revealed a turbid and straw-colored fluid and collapsed the sac. Opening the sac revealed the graft, a gelatinous material, and back-bleeding from the wall. After achieving hemostasis, we applied fibrin glue and oxidized cellulose and closed the sac with sutures.
